# Fog, Symbiosis, and Survival: The Ecological Architecture of the Grit Crust From the Atacama Desert Represents a Lichen Holobiome Rather Than a Soil Microbiome

**DOI:** 10.1111/1462-2920.70350

**Published:** 2026-06-18

**Authors:** Patrick Jung, Lina Werner, Rebekah Brand, Laura Briegel‐Williams, Karen Baumann, Guillaume Letendu, Michael Lakatos

**Affiliations:** ^1^ XCEL‐Extreme Cryptogam Ecology Lab University of Applied Sciences Kaiserslautern Kaiserslautern Germany; ^2^ Microbial Architecture University of Applied Sciences Kaiserslautern Kaiserslautern Germany; ^3^ Department of Integrative Biotechnology University of Applied Sciences Kaiserslautern Pirmasens Germany; ^4^ Geolaboratory University of Vechta Vechta Germany; ^5^ Laboratory of Soil Biodiversity Université de Neuchâtel Neuchâtel Switzerland

**Keywords:** Caliciales, *Chroococcidiopsis*, desert, metabarcoding, proteobacteria

## Abstract

Biological soil crusts (biocrusts) fulfil key ecological functions in arid ecosystems, yet their microbiome composition remains insufficiently resolved. Here, we characterise the microbial communities of the fog‐dependent grit crust in the Pan de Azúcar National Park (Atacama Desert, Chile) using multi‐marker metabarcoding (16S rRNA, 18S rRNA, ITS2) across 11 coastal–inland sites. Chlorophyll_a+b_ concentrations reached up to 900 mg m^−2^, ranking among the highest reported for arid biocrusts and reflecting exceptional fog‐sustained productivity. Bacterial assemblages were dominated by Proteobacteria and Actinobacteria, fungal communities by lichenized Ascomycota (Caliciaceae), and eukaryotic diversity by the green algal photobiont genus *Trebouxia*. Black‐pigmented crusts with dense colonisation exhibited higher biomass but lower taxonomic richness, consistent with later‐successional, lichen‐dominated stages, whereas lighter, less colonised crusts were taxonomically richer yet functionally less integrated, indicative of earlier succession. The prevalence of *Trebouxia*, lichenized fungi, and lichen‐associated bacterial taxa demonstrates that the grit crust microbiome is structured around symbiotic photobiont–mycobiont interactions rather than typical edaphic microbial assemblages. These findings redefine biocrust paradigms by documenting a fog‐driven, chlorolichen‐based system that bridges the ecological spectrum between lithic lichen communities and conventional soil crusts, establishing a critical baseline for assessing dryland microbial resilience under climate change.

## Graphical abstract

Multi‐marker metabarcoding (16S rRNA, 18S rRNA, ITS2) of the fog‐dependent grit crust in the Atacama Desert reveals a community structured by lichen symbioses rather than typical soil‐derived microbial assemblages. The green algal photobiont *Trebouxia* dominates the eukaryotic community, accompanied by lichenized Caliciales and lichen‐associated Proteobacteria and Actinobacteria, while black crusts represent later‐successional, high‐biomass stages and white crusts earlier, more diverse assemblages. These findings redefine biocrust paradigms by documenting a fog‐driven chlorolichen holobiome that bridges lithic lichens and edaphic biocrusts.

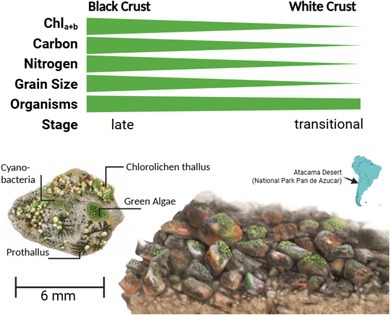

## 1 Introduction

Biological soil crusts (biocrusts) are multifunctional microbial assemblages that occupy the upper millimetres of soil in drylands worldwide (Weber et al. 2022). They consist primarily of cryptogams which are phototrophic microorganisms—cyanobacteria, green algae, lichens, and bryophytes—embedded within a matrix of extracellular polymeric substances (EPS), mineral particles, and heterotrophic bacteria and fungi (Elbert et al. 2012). Despite the microscopic scale of organisms, biocrusts cover up to 12% of the global terrestrial surface and perform crucial ecosystem functions including soil stabilisation, carbon and nitrogen fixation, hydrological regulation, and primary production in otherwise barren landscapes (Rodriguez‐Caballero, Belnap, et al. 2018; Rodriguez‐Caballero, Castro, et al. 2018; Ferrenberg et al. 2015).

The composition and functional attributes of biocrusts vary according to moisture availability, substrate texture, and climatic regime, forming a continuum from early cyanobacterial mats in hyperarid deserts to complex lichen‐ and moss‐dominated crusts in semiarid and temperate zones (Belnap et al. 2016; Corbin and Thiet 2020). In hyperarid regions such as the Negev, Namib, Sahara, and Atacama Deserts, for example, biocrusts are typically low in biomass and C and N content due to extremely low precipitation and high temperatures, with cyanobacteria and algae often sparse (Abed et al. 2019). In deserts like the Mojave or Karoo, greater moisture availability supports higher biomass, especially of cyanobacteria and lichens (Malam Issa et al. 2001) while in semi‐arid regions such as the Colorado Plateau, seasonal moisture pulses enable greater microbial activity and crust development, including hypolithic forms beneath translucent stones (Lee et al. 2016). Similar patterns are observed in central Mongolia, Europe, and Australia, where high biodiversity and dense biomass of lichens and mosses can prevent erosion and enrich soils in nutrients, carbon and/or nitrogen (O'Neill 1994; Chamizo et al. 2012; Samolov et al. 2019). In sub‐humid zones, grazing and fire often lead to cyanobacteria‐dominated crusts (Siebert and Dreber 2019; Szyja et al. 2019). Even in polar and alpine regions, biocrusts persist despite extreme conditions, though productivity is low (Williams et al. 2017).

The development of biocrusts follows a successional sequence beginning most often with filamentous cyanobacteria (e.g., *Microcoleus vaginatus* ) that excrete EPS, binding soil particles and initiating stabilisation (Xiao et al. 2023). Subsequent colonisation by green algae, lichens, and mosses increases structural complexity and enhances biogeochemical cycling. Beyond their ecological functions, biocrusts contribute substantially to ecosystem services at landscape and global scales. They enhance infiltration and reduce erosion by physically stabilising soil aggregates (Kidron et al. 2015; Chamizo et al. 2016). Their phototrophic components drive carbon sequestration and nitrogen fixation, providing organic inputs that sustain heterotrophic communities and pioneer vascular plants (Elbert et al. 2012; Abed et al. 2019). However, biocrusts are vulnerable to mechanical disturbance and climate change. Their recovery after disruption—for example by trampling, vehicles, erosion or weather conditions—can take decades to centuries, particularly in hyperarid regions where growth conditions are marginal (Weber et al. 2016a, 2016b). Understanding the baseline diversity and structure of undisturbed communities is therefore essential to interpret successional trajectories and resilience mechanisms. Biocrusts are also excellent model systems for studying biodiversity—ecosystem function relationships. Their small spatial scale, clear functional attributes, and experimental accessibility make them ideal for linking community composition to ecosystem processes. These diverse ecological functions and services underscore the global significance of biocrusts, particularly in regions where vascular plant cover is sparse and environmental conditions are extreme. This is nowhere more evident than in the Atacama Desert, where unique adaptations have enabled the development of highly specialised biocrust communities under some of the harshest conditions. The Atacama Desert in northern Chile is among the oldest and driest regions on Earth, characterised by annual precipitation below 13 mm and an almost complete absence of rain across extensive areas (Rundel et al. 1991). Yet, along the coastal fog belt, persistent *camanchaca* called fog provides a critical non‐precipitation moisture source that sustains unique fog‐dependent ecosystems (Lehnert et al. 2018). Within the National park Pan de Azúcar (25°–26° S), these conditions support the formation of a so‐called *grit crust*: a biocrust community inhabiting coarse‐grained, granitoid substrates dominated by lichenized green algae and their fungal partners (Jung, Baumann, Lehnert, et al. 2020).

The grit crust differs markedly from the cyanobacteria‐based biocrusts typical of other deserts. For example, their dark (‘black’) and pale (‘white’) surface variants represent contrasting states of microbial colonisation and biomass density (Jung, Baumann, Lehnert, et al. 2020). Black crusts exhibit a dense phototrophic cover, high chlorophyll_a+b_ concentrations, and visible lichen thalli dominated by the green algal photobiont genus *Trebouxia* within fungal taxa such as *Caliciales* (Jung, Brand, et al. 2024; Jung et al. 2025). White crusts, in contrast, display sparse pigmentation and reduced microbial biomass, often comprising early successional assemblages of free‐living algae and saprotrophic fungi (Jung, Brand, et al. 2024; Jung et al. 2025).

Despite intensive taxonomic work on lichen symbionts (Jung, Brand, et al. 2024) as well as local, cultivatable free‐living fungi and microalgae (Jung et al. 2025), the broader microbial community of the Atacama grit crust remains poorly characterised. Such information on the composition and functional roles of accompanying microbial guilds is critical for elucidating nutrient cycling and community assembly under extreme moisture limitation over space and time.

This becomes especially interesting when the interplay between fog deposition and landscape topography along the Atacama coast generates steep environmental gradients in moisture, temperature, and substrate properties across only a few kilometres. In Pan de Azúcar chlorophyll_a+b_ concentrations of the grit crust strongly vary, but reach averages of ~270 mg m^−2^ and maxima up to 900 mg m^−2^, exceeding those of many other deserts (Kidron et al. 2015; Jung, Brand, et al. 2024). These exceptionally high values reflect large standing stocks of phototrophic biomass supported only by fog‐derived water.

Metabarcoding surveys in similar arid habitats reveal that bacterial communities are often dominated by Actinobacteria, Proteobacteria, and Firmicutes—taxa associated with desiccation tolerance, organic‐matter turnover, and stress resilience (Amaresan et al. 2020; Tang et al. 2021; García‐Carmona et al. 2022). Cyanobacteria, while central in many biocrusts, can be surprisingly scarce in fog‐dominated environments where chlorolichens outcompete them once sufficient moisture supports symbiotic photobionts (Kidron 2019). Fungal communities in these systems tend to include lichenized Ascomycota that recycle organic residues (Weber et al. 2022). Endospore‐forming *Bacillus* species and filamentous Actinobacteria (e.g., *Streptomyces*, *Modestobacter*) are known pioneers of desiccation‐tolerant biocrusts, whereas nitrogen‐fixing Proteobacteria may complement the limited heterocytous cyanobacterial contribution (Abed et al. 2019; Weber et al. 2022). Understanding how these taxa assemble under natural, undisturbed conditions provides a baseline for assessing resilience to future perturbations, but traditional cultivation methods capture only a minor fraction of environmental microbial diversity (Jung et al. 2025).

By combining multi‐marker metabarcoding (16S rRNA, 18S rRNA, ITS2) with measurements of chlorophyll_a+b_, carbon, nitrogen, and soil texture, we (i) characterise the taxonomic composition and diversity of bacterial, eukaryotic, and fungal communities across 11 fog‐influenced and arid inland sites; (ii) compare the structural and functional traits of 11 pairs of high‐biomass (blackish) and low‐biomass (whitish) crust sites; and (iii) identify the environmental factors underpinning these differences.

Based on previous observations of the grit crust ecosystem and the fog‐driven environmental gradient along the Atacama coast, we formulated three working hypotheses: (H1) The microbial community of the grit crust is structured primarily by lichen symbioses, resulting in dominance of the green algal photobiont *Trebouxia*, lichenized fungi, and lichen‐associated bacterial taxa rather than a typical soil microbiome dominated by free‐living cyanobacteria. (H2) Black crusts represent later‐successional, high‐biomass communities characterised by lower taxonomic richness but stronger functional integration compared with the more diverse but less developed white crusts. (H3) Environmental gradients related to fog influence and substrate characteristics contribute to spatial variation in microbial community composition across the coastal–inland transect. By characterising the baseline microbial community structure and environmental context of the Atacama grit crust, this study provides a critical reference point for understanding biocrust resilience, succession, and ecosystem functioning in one of the world's most extreme environments.

## 2 Experimental Procedures

### 2.1 Site Description

National Park Pan de Azúcar, established in 1985, spans approximately 437.54 km^2^ along Chile's coastal Atacama Desert, encompassing both the Antofagasta and Atacama regions. The park's landscape is characterised by marine terraces and coastal cliffs, shaped by climatic influences, sea‐level changes, and tectonic uplift. The region experiences an arid climate with minimal annual rainfall, often less than 10 mm (Thompson et al. 2003). However, the presence of camanchaca, a coastal fog, plays a crucial role in sustaining the park's unique ecosystems by providing essential moisture (Lehnert et al. 2018). The park is home to over 20 species of cacti, predominantly from the genus *Copiapoa* (Larridon et al. 2015). These species have adapted to the harsh desert conditions, often relying on fog for moisture. Additionally, the park's flora includes a significant number of endemic species, with 76% of its plant life being unique to Chile (Contreras et al. 2020).

The park supports a variety of wildlife, including guanacos ( *Lama guanicoe* ), culpeo foxes ( *Lycalopex culpaeus* ), and marine species such as the South American sea lion ( *Otaria flavescens* ) and Humboldt penguins ( *Spheniscus humboldti* ).

### 2.2 Plot Establishment and Sampling

**FIGURE 1:**
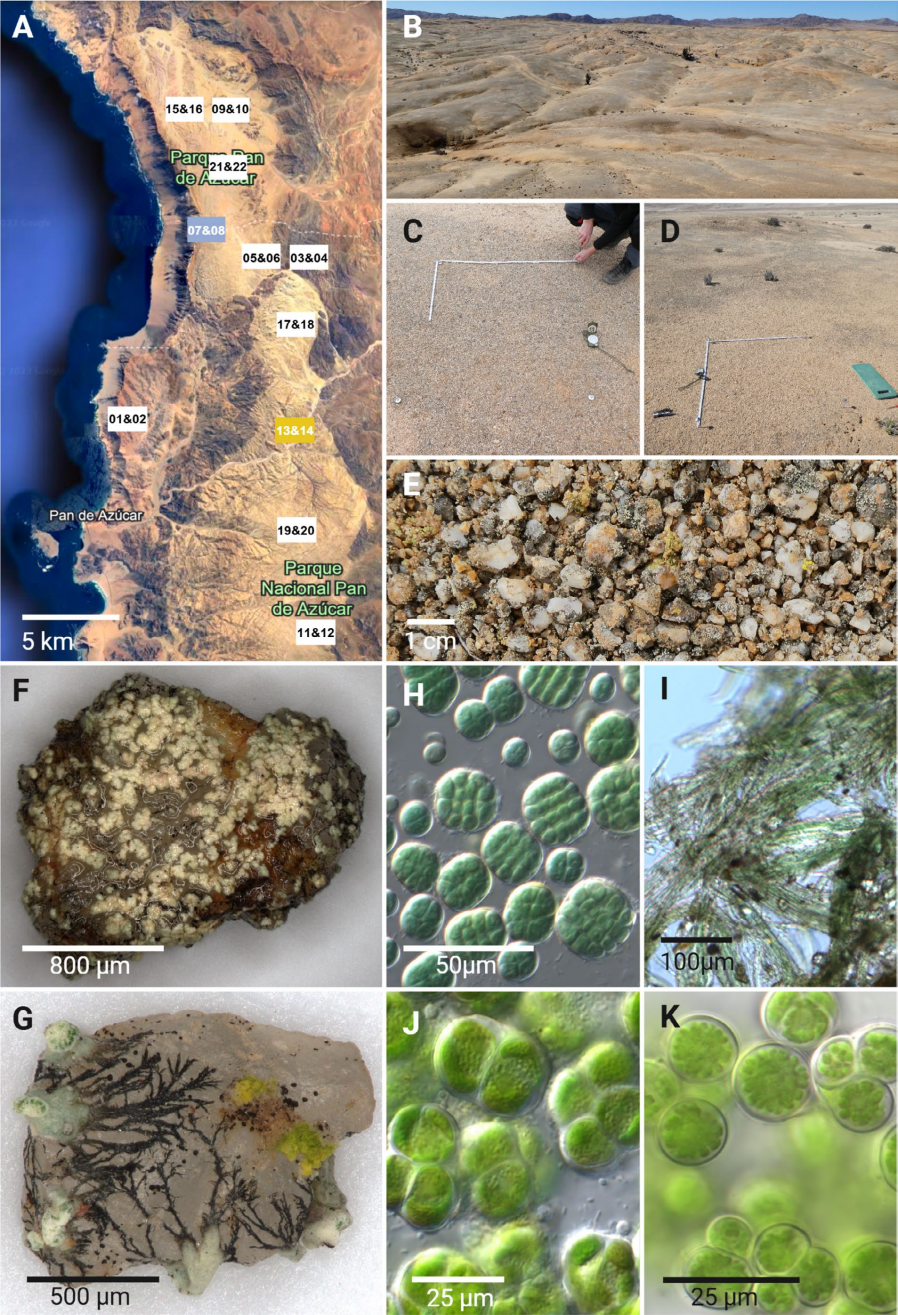
Landscape of the grit crust environment and biocrust target organisms. (A) Map of the National Park Pan de Azucar showing the location of all black (uneven numbers) and white (even numbers). The most humid (07/08, blue) and dry site (13/14, yellow) is marked. (B) Checkerboard pattern of the landscape indicating black (dense microbial colonisation) and white (weak microbial colonisation) areas. (C) Black plot. (D) White plot. (E) Close‐up photograph of the grit crust. (F, G) Close‐up of single grit stones covered with lichens. (H, I) Cyanobacteria; (H) unicellular Chroococcidiopsidales, (I) filamentous *Kastovskya* sp. (J, K) green algae; (J) *Pleurastrosarcina* sp., (K) lichen photobiont *Trebouxia* sp.

Within the National Park Pan de Azúcar, 11 representative sites were selected with extended grit crust coverage (Figure 1A–D). The region is covered by a huge surface mosaic of different soil texture spots next to each other, visible by different surface colours and with unknown origin or explanation. At each site, two plots of one m^2^ were established by marking the corners with 12 cm long metal screws within either densely colonised blackish appearing areas (hereafter referred to as ‘black’) or in a less colonised whitish appearing patch (hereafter referred to as ‘white’) with a maximum of 3 m between both plots. The plots were arranged according to the four cardinal directions using a compass.

Samples for metabarcoding analyses were collected from a 10 cm^2^ subplot within each black and white plot using a sterile metal spatula by collecting the first top cm in a 7 mL plastic tube. Directly after sampling the tube was filled with LifeGuard Soil Preservation Solution (Qiagen, Hilden, Germany). In addition, the top centimetre of another 10 cm^2^ area was removed with a sterile brush and collected in plastic tubes for chlorophyll determination. The same process was repeated at a third 10 cm^2^ subplot for carbon and nitrogen analyses. Outside of the plots but at a maximum of 3 m, 500 g of topsoil material from white and black areas was collected using a brush to estimate the grain size distribution.

### 2.3 Chlorophyll Extraction, Carbon‐ and Nitrogen Content and Grain Size Distribution

In order to estimate the chlorophyll_a+b_ content of the grit crust, an extraction was carried out using the dimethylsulfoxide (DMSO) method (Ronen and Galun 1984), which has been shown to be most suitable for biocrust samples (Caesar et al. 2018).

For total element analyses, samples were ground to < 0.5 mm using a powder mill (Retsch, Haan, Germany) with steel beads (1 and 2.3 mm diameter) at 30 Hz for 15 min. Total C and N were measured in duplicates using a CN‐Analyser (EA3100‐Dual, EuroVector srl, Pavia (PV), Italy). No acid pretreatment was applied prior to analysis. Values therefore represent total carbon including both organic and inorganic (carbonate) fractions. Texture (grain size distribution) was determined by dry sieving after Blume et al. (2010), using a stacked series of sieves with mesh sizes of 2, 3.15, 4, 5, and 6.3 mm. No dispersion agent was applied. Material passing through the smallest sieve (< 2 mm) and material retained above the largest sieve (> 6.3 mm) were collected as the finest and coarsest fractions, respectively.

### 2.4 DNA Extraction, Amplification and Sequencing of Metabarcoding Samples

Prior to DNA extraction, the LifeGuard solution was removed by centrifuging the samples at 2500 g for 5 min and discarding the supernatant. Due to colonisation also of inner structures of the grit stones, the samples were homogenised by beat‐beating at 30 Hz for 5 min with steel beads (1 mm and 2.3 mm diameter) and a powder mill (Retsch, Haan, Germany) according to the DNeasy PowerSoil kit (Qiagen, Hilden, Germany), which was used for DNA extraction. To enhance the yield of extracted DNA, each sample was split into three pseudoreplicates used for DNA extraction. Afterwards, they were combined again and the DNA yield was measured using a Nanodrop One (Thermofisher Scientific, Waltham, USA).

The V4 region of the 16S rRNA of bacteria was amplified using the primer pair F515/R806 (Parada et al. 2016) for bacteria (~300–350 bp) while the universal eukaryotic V4 primer pairs TAReuk454FWD1/TAReukRev3 (Stoeck et al. 2010) were used for the 18S rRNA gene (~390–430 bp) of eukaryotes. The ITS2 (~300–400 bp) region for fungi was amplified by using the primer pair ITS3_KYO2/ITS4 (Toju et al. 2012). The PCRs were performed according to the conditions for each gene region, and library preparation followed the Tagsteady protocol (Carøe and Bohmann 2020). PCR amplification, library preparation and sequencing were carried out by Fasteris (Plan‐les‐Ouates, Switzerland). Sequencing was performed on an Illumina MiSeq platform (2 × 300 bp).

The full bioinformatic analyses were automatically conducted using DeltaMP v0.6 (Lentendu 2023) on a high‐performance computer cluster (‘Centre de Calcul de la Faculté des Sciences’, University of Neuchâtel, Switzerland). Raw Illumina reads were demultiplexed using cutadapt v4.1 (Martin 2011), which assigns sequences to the correct samples based on their unique barcode sequences. Up to two mismatches were allowed in 8 bp barcodes, which were followed by 2 to 4 nt heterogeneity spacers to improve sequencing quality. Cutadapt also removed primer sequences from the 5′ end of reads, allowing a limited number of mismatches (up to 2 for 16S, 3 for 18S and 5 for ITS). Mismatch thresholds were selected based on primer properties, literature precedent (Toju et al. 2012; Haider et al. 2024), and empirical tests to balance sensitivity and specificity during primer removal.

Reads were trimmed from the ends to remove low‐quality base calls, using an adaptive algorithm that ensured retention of at least 75% of high‐quality demultiplexed reads while keeping the expected error rate < 2 and maintaining sufficient read length for reliable paired‐end assembly. Each orientation (R1 or R2) was trimmed to specific lengths optimised for the respective gene region. In the 16S rRNA dataset, forward orientation libraries were trimmed at 280 (R1) and 240 nt (R2) and reverse orientation libraries were trimmed at 260 (R1) and 270 nt (R2). In the 18S rRNA dataset, forward orientation libraries were trimmed at 265 (R1) and 190 nt (R2) and reverse orientation libraries were trimmed at 270 (R1) and 225 nt (R2). In the ITS dataset, forward orientation libraries were trimmed at 260 (R1) and 170 nt (R2) and reverse orientation libraries were trimmed at 235 (R1) and 180 nt (R2). Based on the trimming lengths of paired‐end reads, the amplified ITS2 fragments had an approximate length of 400–430 base pairs.

Amplicon Sequence Variants (ASVs) were inferred using Divisive Amplicon Denoising Algorithm 2 (DADA2) v1.26 (Callahan et al. 2016), which models and corrects sequencing errors to resolve exact biological sequences instead of clustering similar reads into Operational Taxonomic Units (OTUs). This denoising algorithm generates ASVs by applying a statistical error model that distinguishes sequencing errors from true biological variation, offering higher taxonomic resolution and accuracy. Error models were built separately for each primer orientation. After error correction, paired reads were merged (minimum 10 bp overlap, max 1 mismatch); reverse orientation libraries ASVs were reverse complemented using the R package seqinr v4.2–16 (Charif and Lobry 2007) and combined to forward orientation libraries ASVs. All analyses were conducted in R v4.1.2 (R Core Team 2021).

The presence of chimera—artefacts from PCR—was double‐checked first with the DADA2 function removeBimeraDenovo and with the UCHIME de‐novo algorithm as implemented in VSEARCH v2.22.1 (Edgar 2010; Rognes et al. 2016).

Following ASV inference, taxonomic classification was performed using reference databases tailored to each marker gene: SILVA (Quast et al. 2012) for the 16S rRNA, PR2 v5.0.0 (Guillou et al. 2013) for the 18S rRNA, and UNITE v9.0 (Abarenkov et al. 2024) for the ITS sequences. The taxonomy was assigned to each ASV using the VSEARCH option ‘usearch_global’. When multiple database entries showed equal sequence similarity to a given ASV, a consensus taxonomy was assigned based on the lowest taxonomic rank for which at least 60% of the best matches agreed. Additional manual comparisons and adjustments to the taxonomical assignments were conducted based on a previous study on isolated green algae, cyanobacteria and fungi from the grit crust of which full 16S rRNA, 18S rRNA and ITS1,2 sequences were generated (Jung et al. 2025). This allowed for a refined taxonomic assignment based on a living culture collection from the same habitat and eradicated artefacts occurring from understudied or not yet sequenced organisms from the Atacama Desert. Corrections were applied by replacing the original database‐assigned taxonomy of each matching ASV with the curated assignment, propagating changes consistently across all downstream analyses. In total, 13 taxonomic corrections were made, affecting 1638 ASVs (13.4% of all ASVs across the three marker datasets) and approximately 2.1 million reads (16.6% of total reads). The majority of corrected reads originated from the ITS dataset (1378 ASVs, 28.3% of ITS ASVs; 26.7% of ITS reads), followed by the 18S dataset (199 ASVs, 18.8% of 18S ASVs; 15.0% of 18S reads) and the 16S dataset (61 ASVs, 1.0% of 16S ASVs; 0.8% of 16S reads). Full details of each correction, including original and final taxonomic assignments, accession numbers, and justifications, are provided in Table S1.

Post‐classification filtering steps were applied to refine the datasets: For 16S, ASVs with < 70% identity to any reference sequence, fewer than 5 reads, or presence in only one sample were excluded, as were sequences identified as chloroplasts. For 18S, ASVs assigned to Metazoa, Fungi, and Embryophytes were removed, alongside those with < 70% identity, < 5 reads, and occurrence in only one sample (Jung, Brand, et al. 2024). For ITS, the identity threshold was set to 60%, and ASVs with < 5 reads, present in only one sample, or assigned to Chlorophyta were removed to focus on fungal communities (Jung, Brand, et al. 2024). Non‐target amplification is common due to conserved ribosomal regions shared among eukaryotic lineages.

The taxonomic assignments based on the above‐mentioned databases (SILVA, PR2, UNITE) were manually checked based on NCBI GenBank (Sherry et al. 2001) blast searches for some selected sequences. In some cases, phylogenetic reconstructions for ASVs representing specific genera were calculated including sequences downloaded from NCBI GenBank. Alignments were prepared using the Muscle algorithm in the program Mega X v11 (Edgar 2004; Kumar et al. 2018) and phylogenetic trees were calculated as described in Jung, Briegel‐Williams, et al. (2024) in order to correct the taxonomic assignment of such metabarcoding derived data (Table S1).

Metabarcoding data were submitted to the European Nucleotide Archive (ENA) under the project code PRJEB72845.

### 2.5 Statistical Analyses

Abiotic parameters were statistically compared and visualised using SigmaPlot v15.1 (Systat Software 2025). All other downstream analyses were performed in R v4.1.2 (R Core Team 2021), and MicrobiomeAnalyst.ca (Dhariwal et al. 2017; Chong et al. 2020) with tailored workflows for each of the three marker regions: 16S rRNA, 18S rRNA, and ITS2. Although the same analytical structure was applied across all datasets—including diversity metrics, ordination, and differential abundance—data‐specific choices were made regarding the optimal taxonomic resolution. Based on initial data exploration, a phylum‐level aggregation for 16S, genus‐level for 18S, and order‐level for ITS2 data was used. This decision reflects the varying taxonomic depth and accuracy achieved per marker in this dataset and was made based on our data‐driven judgement. To read the data, the following R packages were used: readxl (Wickham and Bryan 2015), and readr (Wickham et al. 2015).

To visualise dominant community members, stacked barplots of relative abundances were generated, aggregated at marker‐specific taxonomic ranks. Only the top 10–15 taxa per region were retained for clarity; all others were grouped under ‘Others’. Visualisation was done using R packages ggplot2 (Wickham 2016) and ggpubr (Kassambara 2016), with labels adjusted via ggrepel (Slowikowski 2016). To additionally illustrate the absolute abundance of dominant taxa, a barplot on total read counts was created for the 16S and 18S using ggplot2. Due to large disparities between taxa, ggbreak (Xu et al. 2021) was employed to insert a y‐axis break, improving the readability of less abundant groups in the 18S dataset.

Alpha diversity was estimated using the Chao1 richness estimator, calculated with the vegan v2.5–7 package (Oksanen et al. 2021). To avoid overestimation caused by intraspecific variability—that is, sequence differences within the same biological species due to sequencing artefacts or microdiversity—ASVs were aggregated to the genus level prior to richness estimation. For example, in taxa such as *Trebouxia*, multiple ASVs may represent a single biological species (Jung, Brand, et al. 2024). Bootstrapped Chao1 estimates with standard deviations were calculated and visualised as scatter plots with error bars per sample using ggplot2. To compare community richness between environmental conditions (black vs. white), a paired Wilcoxon signed‐rank test was performed. Abiotic parameters between paired black and white plots were compared using paired Wilcoxon signed‐rank tests.

Community composition of beta diversity was assessed using non‐metric multidimensional scaling (NMDS) based on Bray–Curtis dissimilarity, with dissimilarity matrices calculated via vegan. Count data were normalised to relative abundances prior to ordination. 95% confidence ellipses per group (black vs. white) were plotted, and group differences were tested using PERMANOVA (adonis2, vegan). To identify community members contributing most to observed group differences, Similarity Percentages (SIMPER) analysis (simper, vegan), a non‐parametric method that decomposes Bray‐Curtis dissimilarities into contributions by individual taxa (Clarke 1993), was used. Permutation‐based pseudo *p*‐values were calculated to assess the robustness of these contributions.

Principal Component Analysis (PCA) was conducted on Hellinger‐transformed ASV matrices (decostand, vegan) to explore variation in community structure. To identify environmental drivers, Redundancy Analysis (RDA) was performed with the ‘*envfit*’ function from vegan. The environmental matrix included distance to coast and grain size fractions. The threshold of 4 mm was chosen based on the size distribution data: the third smallest fraction ends at 4 mm, while the next larger fraction starts at > 4 mm, effectively separating smaller and larger grain size. This threshold thus represents a natural midpoint in the dataset and was based on data structure.

To complement the above analyses, heat trees were generated in the web platform MicrobiomeAnalyst for all three marker regions (Dhariwal et al. 2017; Chong et al. 2020). These visualisations provided a taxonomic overview of differential abundances and compositional changes across taxonomic hierarchies.

Ecological functions were assigned for fungi primary lifestyle traits by consolidating the FungalTraits database (Põlme et al. 2020), while for green algae and cyanobacteria literature was used and AlgaeBase (Lefler et al. 2023; Guiry and Guiry 2025). In cases where different sources yielded conflicting functional classifications for a given taxon, assignments were made conservatively by prioritising the most recent or widely accepted reference, or by indicating ambiguous or multiple potential functions. Where no consensus could be reached, taxa were either excluded from functional summaries or marked as uncertain.

## 3 Results

### 3.1 Environmental Conditions Across the Coastal‐Inland Transect

**FIGURE 2:**
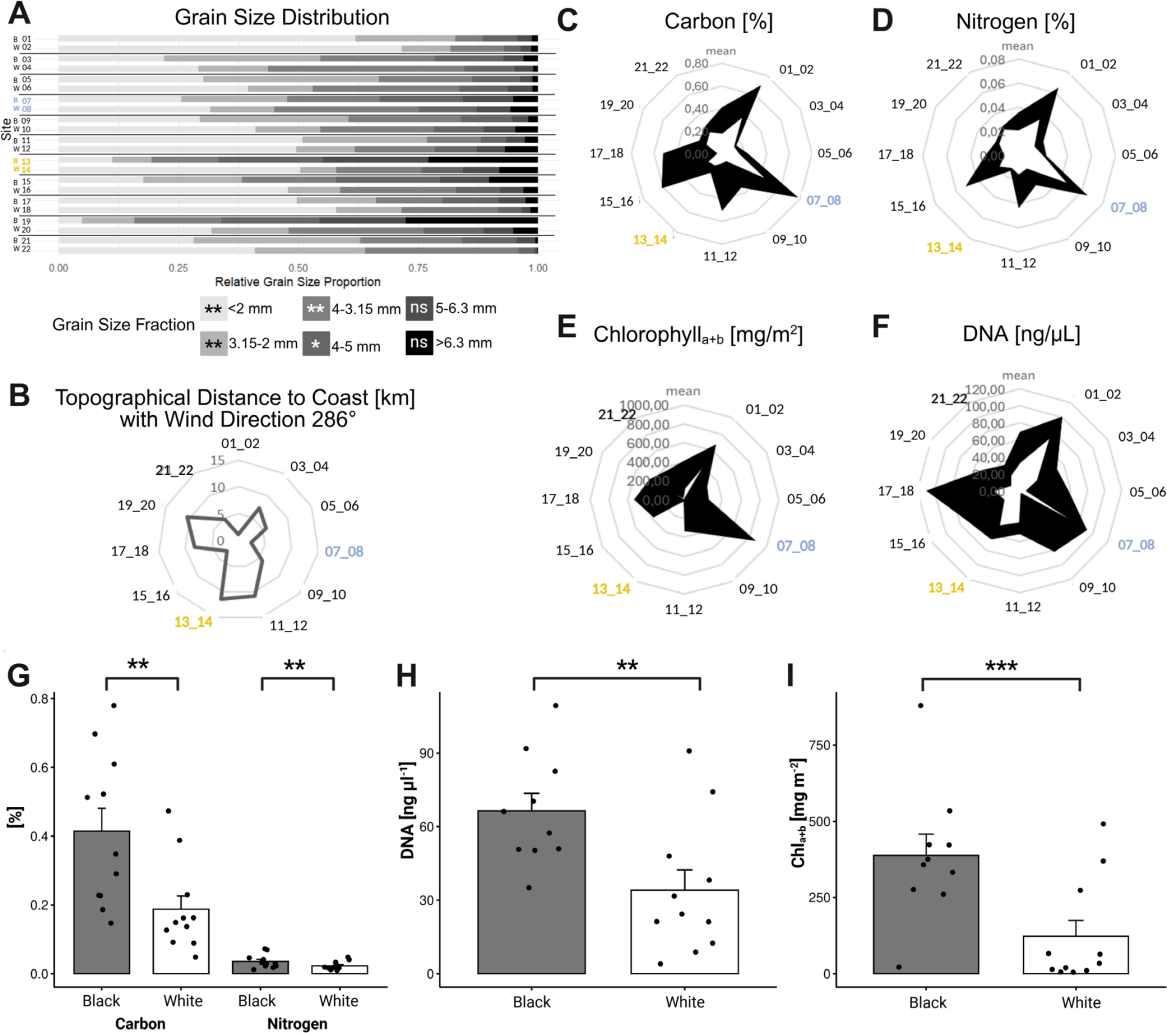
Abiotic plot parameters. (A) Grain size distribution across sites, shown as relative proportions of six grain size classes. Significant differences were found for the fraction < 2 mm (*p* = 0.0028), 3.15–2 mm (*p* = 0.0022), 4–3.15 mm (*p* = 0.0002) and 4–5 mm (*p* = 0.0068). (B) Topographical distance to the coast along a constant wind direction of 286°. (C–F) Environmental characteristics visualised via radar plots, showing: (C) Carbon content (%), (D) Nitrogen content (%), (E) Chlorophyll_a+b_ concentration (mg m^−2^), and (F) total DNA content (ng μL^−1^). Most humid (blue) and dry site (yellow) is marked. (G) Total Carbon‐ (*p* = 0.0017) and Nitrogen content (*p* = 0.0078) of all black versus all white plots indicating significant differences. (H) DNA concentration of all black versus all white plots indicating significant differences (*p* = 0.001). (I) Chlorophyll_a+b_ concentration of all black versus all white plots indicating highly significant differences (*p* = 0.0003). Asterisks indicate significant differences.

The grain size distribution varied markedly between sites (Figure 2A). Besides the two coarse grain size fractions of 5–6.3 mm and > 6.3 mm, all other grain size fractions showed significant differences between black and white plots (Figure 2A). Topographical distance from the coast toward the inland increased progressively, with lowest values for coastal sites (01B/02W, 07B/08W, 15B/16W) and a maximum at sites 13B/14W (Figure 2B).

In general, total carbon‐ (C), nitrogen‐ (N), Chlorophyll_a+b_ content and DNA yield after DNA extraction were higher in each black plot compared to the corresponding white plot (Figure 2C–F). The carbon content was highest at 07B (almost 80%), followed by samples 01B (70%) and 15B (60%). The lowest carbon content in the black samples was at 03B and 13B (~20%). Nitrogen content was again highest in black sites 07B and 01B (> 6%) and lowest in 13B (< 2%). Black crusts showed a higher C:N ratio (10.68) compared to white crusts (6.43).

The chlorophyll_a+b_ concentration was again highest at black site 07B (900 mg m^−2^), followed by 01B (700 mg m^−2^). Highest DNA content was found at site 17B, which was mostly in the middle range for the other parameters (110 ng μL^−1^). Sites 01B and 07B as well as 09B also showed high DNA concentrations between 80 and 100 ng μL^−1^. Low values < 40 ng μL^−1^ were found at sites 03B, 11B and 21B. In the white samples, the maximum value was at 02W and 08W at just under 80 ng/μL, whereas at sites 06W, 10W and 20W the DNA concentration was < 20 ng μL^−1^. The global distribution of total C, N, DNA yield and chlorophyll_a+b_ was significantly higher in black plots than in white plots (paired Wilcoxon signed‐rank test; Figure 2G–I). Effect sizes were large for all parameters (Cohen's *d*: carbon *d* = 1.28, nitrogen *d* = 1.00, DNA yield *d* = 1.38, chlorophyll_a+b_ *d* = 1.65), with black plots showing consistently higher median values: carbon 0.35% (IQR 0.23%–0.61%) versus 0.15% (IQR 0.09%–0.23%); nitrogen 0.03% (IQR 0.02%–0.05%) versus 0.02% (IQR 0.01%–0.03%); DNA yield 66.1 ng μL^−1^ (IQR 50.7–91.8) versus 24.3 ng μL^−1^ (IQR 12.5–48.0); and chlorophyll_a+b_ 375.2 mg m^−2^ (IQR 276.1–534.4) versus 33.9 mg m^−2^ (IQR 10.0–273.8).

### 3.2 Bacterial Community Structure

**FIGURE 3:**
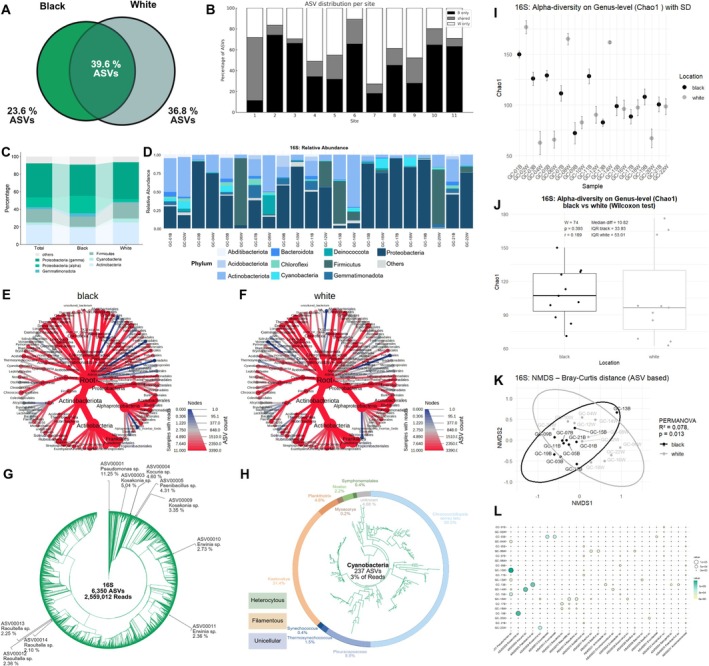
Overview of the 16S rRNA‐based microbial community structure of the grit crust. (A) Venn diagram showing the proportion of unique and shared ASVs between black (high microbial density) and white (lower microbial density) crusts. (B) ASV distribution per site. (C) Compared distribution of dominant bacterial phyla across total, grouped black and grouped white. (D) Relative abundance of phyla per sample. (E, F) Heat trees of 16S rRNA visualising differential taxonomic representation between black (E) and white (F) samples. (G) Radial phylogenetic tree visualising the evolutionary relationships among all 6350 16S ASVs and to highlight the dominant variants based on total abundance. (H) Subtree showing diversity in Cyanobacteria, annotated by relative abundance of the top 10 cyanobacteria ordered according to cyanobacterial growth forms. (I) Alpha Diversity with Chao1 index per sample on genus level. (J) Boxplot comparing alpha diversity on genus level with Chao1 index between black and white by Wilcoxon *t*‐test (*p* = 0.4). (K) NMDS ordination based on Bray‐Curtis distances of ASVs with significant clustering between black and white confirmed by PERMANOVA (*p* = 0.013). (L) Dot plot of core ASVs present across samples, coloured by prevalence; size of the dots reflects the amount of ASV reads.

The final ASV matrices contain 2,559,012 16S reads distributed over 6350 ASVs. According to the actual taxonomic assignments based on the above‐mentioned worldwide databases, 38.6% of all ASVs were globally shared between both crust types, while 23.6% and 36.8% were unique to black and white plots, respectively (Figure 3A). At the local level, an average of 19.0% ± 15.3% of the ASVs were shared between both crust types, while 45.6% ± 22.1% and 35.4% ± 18.0% were unique to black and white plots, respectively (Figure 3B; Table S2).

Comparing the taxonomic composition at the phylum level showed that the bacterial relative abundance of the grit crust consisted of 39% Gamma‐Proteobacteria, 22% Actinobacteria, 15% Firmicutes, 11% Alpha‐Proteobacteria, 2.5% Cyanobacteria, 2% Gemmatimonadota, and the remaining 8% grouped as others (Figure 3C). Differences between black and white samples were mainly caused by a high heterogeneity of the data set due to site‐specific differences: a dominance of Proteobacteria (more Gamma than Alpha) across most samples (excluding samples 06W, 13B, 14W, 20W) and Actinobacteriota was found (Figure 3D). Wherever Actinobacteria had a high relative abundance, the lower was the relative abundance of Proteobacteria (samples 01B, 02W, 08W, 14W) (Figure 3C,D). In black samples, the proportion of Gamma‐Proteobacteria was much higher than in white samples. Therefore, the Actinobacteria percentage in white samples was higher than in black samples (Figure 3C,D).

Differential taxonomic representation on order‐level was visualised using heat trees, which represent the taxonomic distribution including the number of detected ASVs for all black versus all white samples (Figure 3E,F). In general, the weighted taxonomic distribution showed that the bacteriome of black compared to white samples differed only due to a few lineages (Figure 3E,F). The Actinobacteria built the strongest lineage with the highest ASV richness in both sample sets, while there were differences on order level in Proteobacteria: white samples comprised less Micrococcales ASVs in the Alphaproteobacteria, but more Pseudomonadales ASVs in Gammaproteobacteria. For Cyanobacteria there were more Nostocales and Thermosynechoccales ASVs in black than in white, while more Chrooccales ASVs were found in white.

The phylogenetic tree reconstructed from all 6.350 ASVs depicted the community complexity in terms of genotypic diversity and shows the top 10 most abundant ASVs (Figure 3G): ASV0001 *Pseudomonas* sp. represented over 10% of all ASV reads in the dataset, followed by ASV00003 *Kosakonia* sp. with 5% and ASV00004 *Kocuria* sp. with 4.6%. Another *Kosakonia* sp. represented 3.35% of all ASV reads. Each of the three *Raoultella* sp. assigned ASVs 00012, 00013, 00014 made around 2% of reads. In addition, there were two Erwinia sp. ASVs (00010, 00011) with slightly more than 2% each, and one Paenibacillus sp. ASV with more than 4% (Figure 3G). Within Cyanobacteria, 237 ASVs were detected, which accounts for 2.5% of the total ASVs, predominantly belonging to unicellular (~55%) and filamentous, non‐heterocytous (~36%) morphotypes, while heterocytous morphotypes represented the smallest fraction (~5%) (Figure 3H). The most common representatives of the phylum were *Chroococcidiopsis* sensu latu (~50%, unicellular; Figure 1H), followed by *Kastovskya* (~30%, filamentous, non‐heterocytous; Figure 1H) and Pleurocapsaceae (~9%, unicellular).

Alpha diversity for each individual sample, calculated using Chao1 index at genus level, showed a trend toward higher richness in black samples, although this difference was not statistically significant (Wilcoxon *t*‐test, *p* = 0.4; Figure 3I,J). The values of the Chao1 index ranged between 50 (sample 04W) and over 175 (sample 02W).

Beta diversity analysis based on Bray–Curtis dissimilarity showed moderate but statistically significant differences in bacterial community composition between black and white samples (PERMANOVA, *p* = 0.013, *R* ^2^ = 0.078; Figure 3K).

Distribution of the top 25 ASVs showed that each ASV was consistently present across most samples like ASV00001 *Pseudomonas* sp., but there was no recognisable pattern in terms of read counts across the locations indicating a heterogenous data set (Figure 3L).

Redundancy Analysis (RDA) was performed and revealed that among individual environmental predictors, Location (black vs. white) was the only variable that significantly contributed to community variation (*F* = 3.02, *p* = 0.003). All other predictors, including topographical distance to coast (*p* = 0.297), the raw grain‐size fraction (< 2–4 mm; *p* = 0.168), and the grain‐size PCA axes representing general sorting (Grain_PC1; *p* = 0.665) and fine–coarse contrast (Grain_PC2; *p* = 0.338), were non‐significant and are therefore not shown.

### 3.3 Microalgae and Protist Community Structure

**FIGURE 4:**
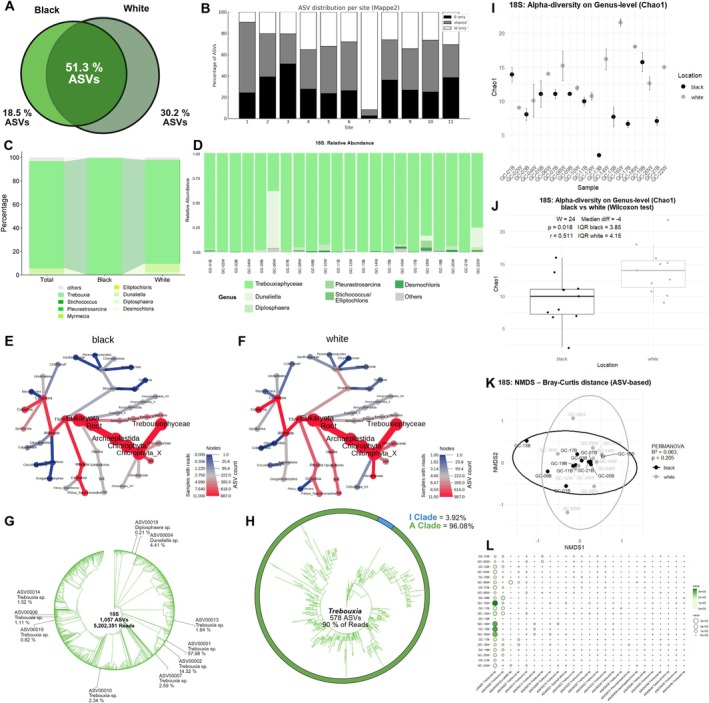
Overview of the 18S rRNA‐based microbial community structure of the grit crust. (A) Venn diagram showing the proportion of unique and shared ASVs between black (high microbial density) and white (lower microbial density). (B) ASV distribution per site. (C) Distribution of dominant eukaryotic phyla across all samples, grouped black and white. (D) Relative abundance of general per sample. (E, F) Heat trees of the 18S rRNA showing differential taxonomic representation in black (E) and white (F) samples, highlighting order‐level shifts. (G) Phylogenetic tree of all 18S ASVs with the 10 most prominent ASVs labelled. (H) Subtree showing diversity in *Trebouxia*, annotated by clade affiliation (A‐ and I‐clade). (I) Alpha diversity with Chao1 index per sample on genus level. (J) Boxplot comparing alpha diversity between black and white samples (Wilcoxon *t*‐test, *p* = 0.019). (K) NMDS ordination based on Bray‐Curtis distances of ASVs with significant clustering between black and white samples (PERMANOVA, *p* = 0.0031). (L) Dot plot of core ASVs across samples, scaled by prevalence.

The final ASV matrices contain 5,202,351 18S reads distributed over 1057 ASVs. A total of 51.3% of ASVs were shared globally between both crust types, while 18.5% and 30.2% were unique to black and white crusts, respectively (Figure 4A). At the local level, an average of 36.6% ± 14.8% of the ASVs were shared between both crust types, while 29.2% ± 12.4% and 32.2% ± 21.1% were unique to black and white plots, respectively (Figure 4B; Table S2).

Comparing the taxonomic composition on genus‐level showed that the eukaryotes of the grit crust consisted of 90% *Trebouxia*, followed by 4% *Dunalliella*, 1% *Diplosphaera* and other green algal genera of which each accounted to less than 1% besides 4% of other ASVs assigned to eukaryotes (Figure 4C). In black samples, the contribution of *Trebouxia* was especially pronounced, while white samples harboured slightly more diverse phyla including *Dunaliella* and *Diplosphaera* (Figure 4C,D).

Differential taxonomic representation on order‐level was visualised using heat trees, which represent taxonomic distribution including the number of detected ASVs for all black versus all white samples (Figure 4E,F). In general, *Trebouxia* showed the strongest enrichment across both black and white, although lineage‐specific differences were observed. For instance, members of Chlorophyceae and *Desmochloris* appeared more frequently in white samples, while Chlorophyta occurred more often in black samples (Figure 4E,F). Three of the four genera from the eukaryotic supergroup Telonemia, Stramenopila, Alveolata, Rhizaria (TSAR) were contained: Rhizaria, which was much more strongly represented in black than in white; Alveolata, in which the ASV counts were distributed differently in black and white; and Stramenopiles, which had only a few ASV counts in both black and white, but slightly more in white. Alveolata included the genus *Colpodea*, which had a high proportion of ASV reads in both black and white relative to the total number of ASV reads. The lineage of Rhizaria was more strongly represented in the number of ASV counts in black than in white (Figure 4E,F).

The 10 most prominent ASVs, of which eight were assigned to different *Trebouxia*, accounted for most of the sequences (Figure 4G). The single ASV00001 *Trebouxia* sp. accounted for almost 58% of all reads. ASV00019 was assigned to Diplosphaera sp. with 0.21% of the reads and ASV00004 representing *Dunaliella* sp. with 4.41%. Within the Chlorophyta, the genus *Trebouxia* alone represented 578 ASVs—over 90% of all reads in the dataset (Figure 4G,H). The genetic diversity within the genus Trebouxia was demonstrated in a phylogenetic tree showing that 96% were assigned to the A‐clade of the genus and 4% were assigned to the I‐clade while representatives from the remaining clades were not detected (Figure 4H).

Alpha diversity, assessed using the Chao1 index at genus level, showed that white crusts were significantly more diverse than black crusts (Wilcoxon *t*‐test, *p* = 0.019; Figure 4I,J). In the 18S rRNA dataset, the related sites of black and white also showed similar Chao1 values to each other compared to the 16S rRNA. The values were close to 0 (sample 13B) up to the highest value of over 20 (sample 16W). The boxplot showed a significantly higher Chao1 index for white compared to black (Figure 4J).

Beta diversity analysis using Bray‐Curtis dissimilarities revealed significant differences in community composition between black and white sites (PERMANOVA, *p* = 0.0031, *R* ^2^ = 0.063; Figure 4K). Black crust samples clustered more closely along the NMDS1 axes, while white crusts were more widely spread on the NMDS2 axes, indicating greater variability.

The distribution of the top 25 ASVs according to the sampling sites showed that while some *Trebouxia* sp. assigned ASVs were present across nearly all samples (ASV00001, ASV00002; 13B excluded), most of the top 25 ASVs were limited to a few sites, underscoring spatial variation in microeukaryotic composition (Figure 4L).

Redundancy Analysis (RDA) was performed and revealed that among individual environmental predictors, Location (black vs. white) was the only variable that significantly contributed to community variation (*F* = 3.02, *p* = 0.002). All other predictors, including topographical distance to coast (*p* = 0.312), the raw grain‐size fraction (< 2–4 mm; *p* = 0.145), and the grain‐size PCA axes representing general sorting (Grain_PC1; *p* = 0.685) and fine–coarse contrast (Grain_PC2; *p* = 0.329), were non‐significant and are therefore not shown.

### 3.4 Fungal Community Structure

**FIGURE 5:**
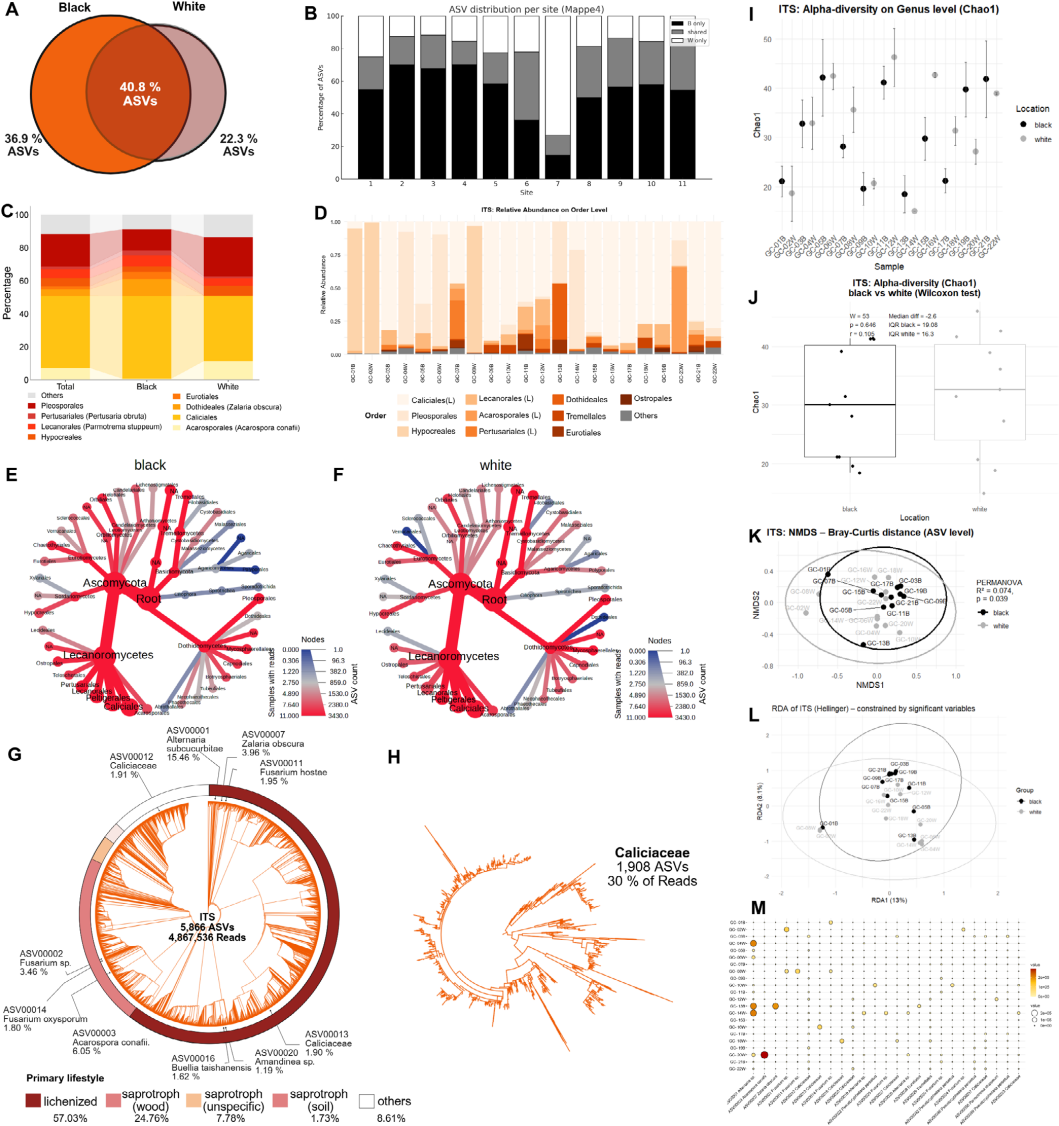
Overview of the ITS‐based fungal community structure in the grit crust. (A) Venn diagram showing the proportion of unique and shared fungal ASVs between black (high microbial density) and white (lower microbial density) crusts. (B) ASV distribution per site. (C) Stacked bar plot comparing the dominant fungal orders across all, grouped black and grouped white samples. (D) Relative abundance of fungal order per sample; (L) means lichenized. (E, F) Heat trees of the ITS visualising differential taxonomic representation between black (E) and white (F) samples, highlighting shifts in major fungal lineages. (G) Phylogenetic tree of all ITS ASVs with the 10 most abundant ASVs labelled; the pie chart shows the percentage distribution on order‐level of the primary lifestyle (based on fungal traits). The category saprotroph (unspecific) included saprotrophic fungi with confirmed decomposer function, but lacking clear substrate affiliation (e.g., wood, dung, litter), whereas saprotrophic (wood) denoted taxa specialised in lignocellulosic degradation. (H) Subtree showing diversity in Caliciales. (I) Alpha diversity with Chao1 index per sample at genus level. (J) Boxplot comparing genus‐level alpha diversity between black and white samples (Wilcoxon *t*‐test, *p* = 0.7). (K) NMDS ordination based on Bray‐Curtis distances, showing significant differences between black and white fungal communities (PERMANOVA, *p* = 0.039). (L) Reduced RDA model with distance to coast (*p* = 0.018) as significant predictor and grain size as non‐significant predictor (*p* = 0.671). (M) Dot plot of core fungal ASVs across samples, with dot size and colour indicating prevalence.

The final ASV matrices contain 4,867,536 ITS reads distributed over 4866 ASVs. According to this, a total of 40.8% of ASVs were shared between both crust types, while 36.9% and 22.3% were unique to black and white samples, respectively (Figure 5A). At the local level, an average of 24.1% ± 8.9% of the ASVs were shared between both crust types, while 53.7% ± 16.3% and 22.2% ± 17.5% were unique to black and white plots, respectively (Figure 5B; Table S2).

Comparing the taxonomic composition on order‐level showed that the fungal community of the grit crust consisted of 44% Caliciales (lichenized), 20% Pleosporales, 7% Acarosporales (lichenized), 5% Lecanorales (lichenized), 5% Hypocreales, 4% Dothideales, 2% Eurotiales, 2% Pertusariales (lichenized), and the remaining 11% grouped as others (Figure 5C). Differences within the fungal communities between black and white samples were mainly caused by a high heterogeneity of the data set due to site‐specific differences (Figure 5C,D): Black samples harboured more Caliciales and Dothideales, while Pleosporales were more represented in white samples (Figure 5C). The relative abundance highlighted shifts in dominant orders across individual samples, especially with a low abundance of Caliacales in the sample pair 01B/02W compared to the most other samples (Figure 5D). The sample pair 01B/02W looked likely the same with a high dominance of Hypocreales.

Differential taxonomic representation on order‐level was visualised using heat trees, which represent the taxonomic distribution including the number of detected ASVs for all black versus all white samples (Figure 5E,F). Here, the Caliciales represented the group with the most reads for both sample types. In addition, overall higher diversity in black samples could be shown for Lecanoromycetes with Acarosporales, Caliciales, Lecanorales and Pertusariales in black samples, while the Basidiomycota had more ASV counts in white samples. In the group of Dothideomycetes, Dothideales and Phaeothecales were more strongly represented in black, while Neophaeothecales had more ASV counts in white. Lichenostigmatales, Eurotiales and Hypocreales showed similar ASV counts in both black and white, while Candelariales were more strongly represented in black (Figure 5E,F).

The ITS phylogenetic tree revealed 5876 ASVs comprising nearly 4.9 million reads (Figure 5G). Among the top ten ASVs, *Alternaria subcurbitae* (ASV00001) dominated with 15.46% of total reads, followed by the lichen mycobiont *Acarospora conafii* (ASV00003) with 6.05% and *Zalaria obscura* (ASV00007) with 3.96%. Other notable taxa included two Caliciaceae, *Fusarium hostae*, *Fusarium* sp., and *Fusarium oxysporum* (each ~2%–3%) and one ASV of each lichen mycobionts *Amandinea* sp. and *Buellia taishanensis*. Together, the top 10 ASVs accounted for ~40% of all ITS reads. The functional classification of fungal orders (Figure 5G) revealed a clear dominance of lichenized taxa. Most sequences were associated with a lichen‐forming primary lifestyle (~57%), followed by wood saprotrophs (~25%) and smaller proportions of unspecific (~8%) and soil saprotrophs (~2%). Additional lifestyles such as litter saprotrophs, plant pathogens, root endophytes, animal and lichen parasites contributed only marginally and were summarised in others, also the functionally unassigned ASVs (likely due to taxonomic uncertainty or functional ambiguity). A subtree focused on Caliciaceae showed a high genotypic diversity of ASVs (1986), which together made up ~30% of all ITS reads (Figure 5H).

Alpha diversity, assessed using Chao1 index at genus level, did not show significant differences between black and white sites (Wilcoxon *t*‐test, *p* = 0.7; Figure 5I,J). However, white samples generally exhibited greater variability in Chao1 values, with the highest richness (~45) in sample 12W. Sample 13B again showed the lowest diversity values in black, consistent with the 16S rRNA‐ and 18S rRNA‐derived results. While black and white sample pairs at many locations showed similar alpha diversity, a few sites (e.g., 15B/16W, 17B/18W) showed large differences.

Beta diversity analysis revealed a weak but statistically significant separation between black and white samples based on Bray–Curtis dissimilarity (PERMANOVA, *p* = 0.039, *R* ^2^ = 0.074; Figure 5K). White samples showed broader spread along the NMDS1 axis to the left, while most of the black samples were more condensed in one group, excluding samples 01B and 13B. All black samples were within the ellipse of white. Some sample pairs, such as 05B/06W or 21B/22W, clustered closely, while others, like 13B/14W, did not.

The full RDA model was significant (*F* = 2.04, *p* = 0.013), and axis tests revealed that among environmental predictors the first grain‐size PCA axis representing sediment sorting (Grain_PC1; *F* = 2.54, *p* = 0.041) was significant (Figure 5L). This indicates that ITS communities are influenced by grain‐size sorting (PC1), but not by specific individual grain fractions.

The distribution of the top 25 ASVs according to the sampling sites revealed that they were unevenly distributed across samples (Figure 5M). The most samples showed a deviation from their site pair, for example 07B to 08 W, which was lacking most core ASVs. Also, higher read numbers could be found in white samples compared to their site pairs.

## 4 Discussion

### 4.1 Environmental Gradients and the Exceptional Productivity of the Grit Crust

The grit‐crust community of the National Park Pan de Azúcar represents one of the most biologically active systems yet described within the hyperarid Atacama Desert (Figure 1F–K). Despite extreme moisture limitation, average chlorophyll_a+b_ concentrations of about 270 mg m^−2^, with maximum values up to 900 mg m^−2^ frequently exceeded those reported from other deserts (Kidron et al. 2015), approaching levels typical of mesic biocrusts in temperate ecosystems (Caesar et al. 2018). This remarkable productivity is likely driven by the persistent influence of coastal fog (*camanchaca*), which supplies intermittent yet predictable hydration pulses that sustain phototrophic metabolism (Lehnert et al. 2018; Jung et al. 2019) and infrequent condensation or dew events on the soil surface (Jung et al. 2025). Comparable fog‐driven hotspots have been documented in the Namib fog belt (Azúa‐Bustos et al. 2011; Rundel et al. 1991), where condensed moisture supports localised microbial oases under otherwise hyperarid climates (De los Rios et al. 2022).

Across the coastal–inland gradient, microbial biomass and diversity responded strongly to water availability and substrate characteristics (Figure 2). Sites nearest the coast (e.g., 7/8) exhibited elevated DNA concentrations, higher chlorophyll_a+b_ content, and greater proportions of phototrophs—indicators of active primary production. While these sites were characterised by fine substrate texture, inland sites (e.g., 13/14) showed coarse substrate texture and severely low total carbon, and nitrogen pools (Figure 2). In addition, all tested parameters (carbon, nitrogen, DNA yield and chlorophyll_a+b_ content) were significantly higher in black samples compared to white samples that is consistent with the more pronounced dark surface patterns observed in the field. This contrast might not only be linked to coastal or fog influence, but also appears to be mediated by grain size distribution: white plots contained significantly more fine particles (< 5 mm) than black plots. Grain size can directly shape microbial community composition by altering microhabitat architecture, including pore connectivity, water‐holding capacity, and gas diffusion. A higher proportion of fine particles typically increases capillary water retention and creates smaller, more tortuous pore spaces, which can favour microbial assemblages adapted to longer moisture residence times and stronger oxygen gradients (e.g., facultative anaerobes) while simultaneously constraining filamentous growth forms and reducing the spatial extent of interconnected phototrophic mats. In contrast, coarser substrates often provide larger intergranular voids and a more heterogeneous light and moisture regime, facilitating thicker surface biofilms, deeper microbial penetration, and stable attachment of EPS‐rich communities that are frequently dominated by filamentous cyanobacteria and associated heterotrophs (Rodriguez‐Caballero, Belnap, et al. 2018). These physical differences can feed back on pigmentation and biomass development: higher surface stability and repeated wet–dry cycling in coarser plots may promote the accumulation of photoprotective pigments and dense phototroph–heterotroph consortia, which would be reflected in elevated chlorophyll content, DNA yield, and organic matter pools. Moreover, finer‐grained matrices can enhance mineral–organic interactions and potentially increase sorption of nutrients or salts, which may impose additional selective pressures on community structure and functional traits. Overall, the observed grain‐size effect is compatible with a bioweathering scenario driven by grit crust communities, where microbial colonisation, EPS production, and microbially mediated mineral alteration progressively modify particle size distributions and thereby reinforce distinct community states, as outlined in Jung, Baumann, Emrich, et al. (2020).

### 4.2 Integrating Metabarcoding andCulture‐Derived Taxonomy for Community Reconstruction

A unique strength of this study lies in the combination of high‐throughput metabarcoding and culture‐derived taxonomic data from the same extreme habitat. Metabarcoding alone provides comprehensive insights into the diversity and structure of microbial communities, yet its accuracy critically depends on the completeness and correctness of reference databases (Nilsson et al. 2019; Tedersoo et al. 2014). Many organisms from extreme environments, including the Atacama Desert, remain underrepresented in such databases, leading to taxonomic mismatches, erroneous assignments, and inflated ‘unknown’ sequence fractions (Nissimov 2017; Rippin et al. 2018). The parallel isolation and sequencing of representative cyanobacteria, green algae, and non‐lichenized fungi from the same grit crust system (Jung et al. 2025) therefore provides an essential calibration dataset for environmental amplicons, enabling a direct refinement of taxonomic resolution. For example, 13 manually corrected assignments were conducted here for 1.638 ASVs derived from lichenized fungi, cyanobacteria, and green algae with more than 2 mio. reads (Table S1). This was only possible due to previous Sanger‐sequencing on lichens from the same habitat such as the unique Caliciaceae population (Jung, Brand, et al. 2024), taxonomic work on certain cyanobacteria such as *Hyella* (Jung et al. 2021) or *Aliterella* (Jung, Mikhailyuk, Emrich, et al. 2020) and green algae such as *Pleurastrosarcina* (Darienko et al. 2019).

By linking metabarcoding‐derived ASVs to curated Sanger sequences from living isolates, the dataset achieves several advances: (i) the validation and correction of ambiguous assignments in global reference databases such as SILVA, PR2, and UNITE; (ii) the recovery of previously undescribed or misclassified taxa, particularly within Chroococcidiopsidales and filamentous Actinobacteria; and (iii) the contextualization of sequence abundances with morphological and physiological traits of viable organisms. This polyphasic integration reduces the discrepancy between environmental and culture‐based inventories—a long‐standing limitation in desert microbiology and elsewhere (Epstein 2013; Louca et al. 2016)—and transforms the dataset into a dynamic reference for future updates of metabarcoding pipelines and regional barcode libraries.

Importantly, combining both methodological layers also advances ecological interpretation. The detection of taxa both in environmental amplicons and as living isolates confirms their metabolic relevance within the grit crust holobiome, distinguishing active community members from relic DNA signals (Carini et al. 2016). Moreover, by anchoring ASV identities to characterised isolates, community assembly processes and successional trajectories can be interpreted in functional rather than merely compositional terms. This integrative approach thus establishes a robust framework for resolving the microbial community architecture of extreme biocrusts, offering a model for improving global sequence repositories and enhancing the reproducibility of microbial community studies across understudied ecosystems.

### 4.3 Community Assembly and Successional Stage

The dichotomy between black and white crusts reflects not only differences in biomass and pigmentation but also clear differences in community assembly across the three major organismal groups analysed (bacteria, microeukaryotes, and fungi). These differences are visible in taxonomic dominance, biodiversity patterns, and abiotic parameters and therefore represent the central ecological signal emerging from the (metabarcoding) dataset (Table 1).

**TABLE 1:** Major differences between black and white grit crusts across abiotic parameters and microbial community composition.

Category	Parameter/Community component	Black crust	White crust	Evidence
Abiotic parameters	DNA yield	Higher DNA concentrations after extraction	Lower DNA concentrations	Figure 2F,H.
	Grain size distribution	Higher proportion of coarse particles (> 4 mm)	Higher proportion of fine particles (< 4–5 mm)	Figure 2A.
	Chlorophyll_a+b_	Much higher values (up to ~900 mg m^−2^), indicating dense phototrophic biomass	Lower concentrations	Figure 2E,I.
	Carbon	Higher carbon pools	Lower carbon content	Figure 2C,G.
	Nitrogen	Higher nitrogen concentrations	Lower nitrogen concentrations	Figure 2D,G.
	C:N ratio	Higher C:N ratio	Lower C:N ratio	Figure 2G.
Bacteria (16S)	Dominant bacterial groups	Higher Gammaproteobacteria (*Pseudomonas, Kosakonia, Raoultella*)	Higher Actinobacteria	Figure 3C–D.
	Cyanobacteria	Low abundance (< 3%), mainly *Chroococcidiopsis*	Slightly more frequent but still minor	Figure 3H.
Microeukaryotes (18S)	Dominant phototrophs	Strong dominance of *Trebouxia*	Higher diversity of Chlorophyta (*Dunaliella*, *Diplosphaera*)	Figure 4C–D.
	Alpha diversity	Lower richness	Higher richness	Figure 4I–J.
Fungi (ITS)	Dominant fungal groups	Lichenized fungi dominate (Caliciales, Acarosporales)	Non‐lichenized fungi more common (Pleosporales, Hypocreales)	Figure 5C–D.
	Functional lifestyle	Predominantly lichen‐forming taxa	More saprotrophic/opportunistic fungi	Figure 5G.
Ecological interpretation	Community structure	Integrated lichen‐dominated holobiome	Taxonomically heterogeneous assemblage	See Section 4
	Successional stage	Late successional stage	Early/transitional stage	See Section 4

In the bacterial communities, both crust types were dominated by Proteobacteria and Actinobacteria (Figure 3C–D). However, their relative proportions differed between crust types. Black crusts tended to show higher relative abundances of Gammaproteobacteria, whereas Actinobacteria were more prominent in white crusts (Figure 3C). Members of these groups represent metabolically versatile taxa commonly associated with arid soils and lichen‐associated microbiomes, including genera such as *Pseudomonas*, *Kosakonia*, and *Kocuria* (Grube et al. 2015; Cardinale et al. 2008). Despite these compositional shifts, alpha diversity of the bacterial communities did not differ significantly between black and white crusts (Figure 3I–J), suggesting that bacterial community assembly is influenced more by local microhabitat conditions than by successional stage alone.

In contrast, the microeukaryotic phototrophic community showed the strongest differentiation between crust types. Black crusts were overwhelmingly dominated by the lichen photobiont genus Trebouxia, which accounted for the vast majority of reads in the 18S dataset (Figure 4C–D,G). This dominance resulted in low alpha diversity values in black crusts (Figure 4I–J). White crusts, in contrast, harboured a broader spectrum of green algae including *Dunaliella* and *Diplosphaera*, resulting in significantly higher richness (Figure 4C–D,I–J). These patterns indicate that the phototrophic community of the grit crust transitions from a taxonomically diverse assemblage of free‐living algae to a highly specialised photobiont‐dominated system.

A comparable shift is visible in the fungal communities. Black crusts were characterised by strong dominance of lichenized fungal orders, particularly Caliciales and Acarosporales (Figure 5C–D), which are typical components of lichen holobiomes (Moya et al. 2021; Hawksworth and Grube 2020). White crusts, in contrast, showed higher contributions of non‐lichenized ascomycetes, including Pleosporales and Hypocreales (Figure 5C–D), which are commonly associated with saprotrophic or opportunistic lifestyles (U'Ren et al. 2010). It should be noted that the ITS3_KYO2/ITS4 primer pair is known to underrepresent Glomeromycota and some Basidiomycota, meaning that the contribution of these lineages to white crusts may be underestimated (Toju et al. 2012). These differences were also reflected in the functional classification of fungal lifestyles, where lichen‐forming taxa dominated the fungal dataset overall (Figure 5G).

In addition, the carbon‐to‐nitrogen ratio further reflects the contrasting ecological states of the two crust types. Black crusts exhibited a substantially higher C:N ratio (10.7) than white crusts (6.4), indicating a greater accumulation of organic carbon relative to nitrogen. Elevated C:N ratios are typically associated with increased phototrophic biomass and more developed organic matter pools in biocrust ecosystems. In contrast, the lower ratios observed in white crusts are consistent with early‐stage microbial assemblages dominated by fast‐turnover microbial biomass and limited carbon storage. Together with the dominance of *Trebouxia* and lichenized fungi, these patterns support the interpretation that black crusts represent a more mature and functionally integrated stage of the grit crust ecosystem.

Together, these patterns demonstrate consistent and ecologically interpretable differences in community assembly between the two crust states (Table 1), though the low PERMANOVA *R* ^2^ values (0.063–0.078) indicate that crust type explains only a modest fraction of total variation, with additional factors such as microtopography, historical disturbance, or fine‐scale substrate heterogeneity likely contributing to the overall compositional variance. Black crusts represent structurally cohesive communities dominated by lichen symbioses, whereas white crusts host more taxonomically heterogeneous assemblages of free‐living algae, saprotrophic fungi, and soil‐associated bacteria. These contrasting community structures are consistent with the interpretation that black crusts correspond to later‐successional stages, whereas white crusts represent earlier or transitional community states along the local moisture and stability gradient. Successional dynamics in biocrusts typically begin with microbial pioneers that stabilise the substrate and introduce organic matter (Belnap and Lange 2003; Elbert et al. 2012). Over time, these communities are often replaced by lichen‐dominated assemblages as environmental conditions allow the establishment of stable photobiont–mycobiont partnerships (Bowker et al. 2010; Corbin and Thiet 2020). The dominance of Trebouxia and lichenized fungi in the black crusts therefore suggests that these communities represent a relatively mature state of the grit crust ecosystem.

### 4.4 Functional Differentiation of Black and White Crusts: Lichen‐Dominated Holobiomes

The dominance of *Trebouxia* photobionts and associated lichenized fungi mainly of the Caliciales growing in and around the single grits (Figure 1E–G) defines a tightly integrated symbiotic consortium that functions analogously to a lichen holobiome rather than a biocrust‐soil microbiome (Table 2). In lichen holobiomes, photobionts supply fixed carbon, mycobionts regulate hydration and nutrient exchange, and bacterial and fungal partners contribute to nitrogen turnover and stress tolerance (Moya et al. 2021; Zhang et al. 2023; Rana et al. 2025). The result is a metabolically interdependent, self‐sustaining microecosystem (Hawksworth and Grube 2020). Typical microorganisms detected in lichen holobiomes are for example proteobacteria (e.g., *Pseudomonas*, *Rhizobium*; Grube et al. 2015), actinobacteria (*Streptomyces*, *Kocuria*; Cardinale et al. 2008), firmicutes (*Bacillus*, *Paenibacillus*; Cernava et al. 2015), lichenicolous ascomycota (*Zwackhiomyces*, *Phoma*; Muggia et al. 2013) or and endolichenic fungi (*Penicillium*, *Alternaria*; U'Ren et al. 2010)—all of which were found in significant proportions in the grit crust.

**TABLE 2:** Overview of dominant taxa identified in the grit crust of the Atacama Desert with corresponding habitat preferences and ecological functions.

Taxon/Phylum	Dominant functional traits/Lifestyle	Ecological/Successional role
Actinobacteria	Decomposers; drought and UV tolerance; organic matter cycling (e.g., *Streptomyces*)	Late‐stage resilience; persistent in mature crusts
Cyanobacteria – filamentous	Soil stabilisation; EPS production; carbon and nitrogen input (e.g., *Microcoleus*)	Mid‐successional; ecosystem engineers
Cyanobacteria – heterocytous	Nitrogen fixation (e.g., *Nostoc*)	Late‐successional; key for N input
Cyanobacteria – unicellular	Photosynthesis; UV protection via sheath pigments (e.g., *Chroococcidiopsis*)	Early phototrophic coloniser; pioneer
Firmicutes	Endospore formation; extreme desiccation and salt tolerance; rapid germination (e.g., *Bacillus*)	Pioneer taxon; early coloniser after disturbance
Proteobacteria – Alphaproteobacteria	Symbiosis potential; nitrogen cycling (e.g., *Rhizobium*)	Host‐associated; mutualists in mature crusts
Proteobacteria – Gammaproteobacteria	Fast growth; metabolic generalists; versatile carbon use (e.g., *Pseudomonas*); nitrogen fixation (e.g., *Kosakonia*)	Opportunistic early coloniser; rapid responders
Alveolata	Parasitic or heterotrophic protists	Microbial grazers; influence community composition
Colpodea	Heterotrophic protists	Bacterial predation; nutrient cycling
Desmochloris	Free‐living green algae	Primary production; early colonisation
Diplosphaera	Free‐living or facultative symbiont green algae	Primary production; early colonisation of biocrusts
Dunaliella	Free‐living, halophilic green algae	Osmoregulation; primary production in saline environments
Stichococcus/Elliptochloris	Free‐living green algae	Photosynthesis; soil stabilisation; early biocrust colonisers
Rhizaria	Heterotrophic protists	Bioturbation; microbial loop contribution
Trebouxia	Symbiotic, lichenized photobiont (green alga)	Photosynthesis; carbon for fungal partner; stress resistance; lichen formation
Acarosporales	Lichenized fungi	Pioneer species; extreme aridity/UV tolerance; early soil formation
Basidiomycota	Saprotrophs; lichenicolous fungi	Decomposition; nutrient cycling; interactions with lichens
Caliciales	Lichenized fungi	Soil stabilisation; microhabitat formation; UV protection; slow C fixation; water retention
Eurotiales	Saprotrophs	Rapid, opportunistic decomposition; spore production; early succession
Hypocreales	Saprotrophs/parasites	Soil nutrient turnover; competition
Pertusariales	Lichenized fungi	Long‐lived crust‐formers; soil stabilisation
Pleosporales	Saprotrophs	Organic matter decomposition; nutrient cycling; early coloniser

The reduced ASV richness in black crusts reflects ecological convergence around these dominant symbiotic partnerships. Competitive exclusion and priority effects likely constrain additional colonisation, leading to lower alpha diversity but enhanced stability—patterns widely recognised in mature symbiotic systems (Williams et al. 2017; Corbin and Thiet 2020). Functionally, these lichen holobiome communities perform essential biogeochemical roles, including carbon fixation (via photobionts e.g., *Trebouxia* and epibionts e.g., *Dunaliella*), nitrogen input (via proteobacterial diazotrophs e.g., *Kosakonia*), and mineral surface stabilisation (via fungal hyphae). In contrast, some of the detected microorganisms are likely associated with the surrounding stone or soil matrix rather than the lichen holobiome itself. This is particularly plausible for most detected cyanobacteria, as no lichen‐forming mycobiont known to establish symbioses with cyanobacterial photobionts was identified in the grit crust.

However, the persistence of likely lichen holobiome microorganisms as well as the dominance of *Trebouxia* under hyperaridity highlights symbiosis as a key adaptation to extreme desiccation, analogous to rock‐inhabiting lichens, endolithic‐ or hypolithic consortia (Wierzchos et al. 2015; De los Rios et al. 2022; Azúa‐Bustos et al. 2011).

White crusts, by contrast, exhibit greater taxonomic breadth but lower biomass. Their microbial profiles—enriched in Actinobacteria, non‐lichenized fungi (Pleosporales, Hypocreales), and diverse green algae (e.g., *Dunaliella*, *Diplosphaera*)—suggest a community shaped more by stochastic dispersal and environmental filtering than by established symbioses. The higher variability in alpha diversity indicates dynamic, disturbance‐sensitive assemblages. Similar early‐stage or recolonizing crusts have been described in the Negev and Colorado Plateau, where repeated desiccation–rehydration cycles prevent long‐term stabilisation (Belnap et al. 2003; Lee et al. 2016; Gabay et al. 2022). Functionally, these white crusts likely contribute modestly to nutrient cycling and substrate cohesion but serve as reservoirs for microbial diversity and as potential precursors to lichenized systems. Their presence within close proximity to black crusts underlines the mosaic nature of biocrust succession, where microtopographic variation and localised moisture create patchy mosaics of maturity (Rodriguez‐Caballero, Castro, et al. 2018).

Across both crust types, Proteobacteria and Actinobacteria dominated the bacterial assemblages, reflecting their central role in arid‐soil functionality (Weber et al. 2022; Steven et al. 2018). The abundance of Gammaproteobacteria (e.g., *Pseudomonas*, *Kosakonia*) underscores the prevalence of metabolically versatile taxa capable of rapid response to ephemeral wetting events (Tang et al. 2021). Firmicutes, particularly in inland plots, point to spore‐forming, desiccation‐resistant strategies typical of extreme arid environments (Abed et al. 2019). Fine‐scale taxonomic differentiation among these phyla indicates localised niche specialisation even within apparently uniform habitats, emphasising the need for high‐resolution analyses beyond phylum level (Jung et al. 2025).

The surprisingly low relative abundance of cyanobacteria (< 3%) challenges conventional assumptions about their centrality in desert biocrust function during bio‐geochemical cycles. Oftenly, cyanobacteria are responsible for nitrogen fixation in biocrusts but in the coastal area of the Atacama Desert high proportion of atmospheric nitrogen is transported via fog or dust (González et al. 2011). In addition, for the grit crust, it has been shown that proteobacterial nitrogen fixers (here mainly *Kosakonia*) may compensate for the scarcity of heterocytous cyanobacteria such as *Nostoc*. We note, however, that this remains a hypothesis based on taxonomic identity alone, as no direct evidence of nitrogenase activity (nifH gene expression, metatranscriptomic data, or acetylene reduction assays) was obtained in the present study. Functional validation of diazotrophic activity by *Kosakonia* and related taxa in the grit crust therefore represents an important avenue for future research. Comparable functional redundancy has been observed in hyperarid Namib and Atacama soils, where non‐cyanobacterial diazotrophs contribute significantly to nitrogen input (Hashmi et al. 2020). This replacement underscores how extreme aridity and fog‐based hydration can reshape canonical models of biocrust functioning, referring to the widely accepted conceptual framework of how biological soil crust ecosystems are structured and operate in most drylands.

### 4.5 Comparative Context: Atacama Grit Crust Within Global Biocrust Diversity

Globally, biocrusts exhibit strong biogeographical differentiation driven by climatic water balance and substrate age (Belnap et al. 2003; Pointing and Belnap 2012). Compared with the cyanobacteria‐dominated crusts of most other deserts and drylands, the grit crust of the Atacama represents a distinct functional and compositional endmember. For example, typical lichens found in other dryland biocrusts are lichens such as *Peltula*, *Collema*, or *Psora*, cyanobacteria such as *Microcoleus* or *Nostoc*, and green algae such as *Bracteacoccus* and a diverse set of photobionts often including *Asterochloris* (reviewed in Büdel et al. 2016)—all of which have either not been detected in the grit crust at all or in minor proportions. Whereas other drylands experience periodic rainfall and seasonal regeneration (Hagemann et al. 2015; Kidron 2019), the Atacama's near‐complete absence of precipitation selects for slow‐growing, but frequent fog‐dependent symbioses.

In this sense, the grit crust parallels lithic lichen holobiome communities more than typical soil biocrusts. Their dominance by lichens of the *Caliciales* (Jung, Brand, et al. 2024)—taxa commonly confined to rock or wood substrates (Selva 2013; Piepenbring 2022)—illustrate a unique evolutionary transition from saxicolous (living on rocks) to psammophilous (living on fine grain material such as sand) lifestyles. Such an adaptation may involve morphological and physiological modifications, such as their small thallus sizes, that enable stable thallus formation on loose grits, an ecological innovation not documented elsewhere. This hybrid character blurs traditional boundaries between biocrusts and rock‐inhabiting lichens, expanding the functional diversity of desert cryptogams and highlighting the unique properties of the grit crust.

### 4.6 Ecological and Functional Implications

The coexistence of highly productive, symbiotic black crusts and lower‐biomass, slightly more diverse white crusts across fine spatial scales reveals a dynamic equilibrium between stability and renewal. Black crusts maintain essential ecosystem functions—carbon sequestration, nitrogen input, and substrate stabilisation—under stable microclimatic regimes, while white crusts preserve diversity and colonisation potential in disturbed or marginal niches. Together, they sustain biocrust continuity across the hyperarid landscape.

From a biogeochemical perspective, the substantial chlorophyll_a+b_ stocks and presumed high rates of net primary productivity suggest that these grit crusts contribute meaningfully to carbon fluxes in an environment otherwise characterised by negligible vascular‐plant productivity. Although absolute rates remain unquantified, the close coupling between phototrophic biomass and DNA content implies active metabolic cycling even under extreme aridity (Jung, Brand, et al. 2024). Furthermore, the presence of diazotrophic Proteobacteria indicates a pathway for biological nitrogen input independent of classical cyanobacterial fixation, thereby maintaining essential nutrient turnover.

In ecological terms, the grit crust of the National Park Pan de Azúcar expands current understanding of biocrust diversity by revealing a novel symbiotic configuration—fog‐driven, green‐algal‐dominated, lichenized systems occupying an intermediate successional niche. Their study emphasises the need to consider alternative moisture sources, such as fog and dew, in global models of biocrust distribution and functioning. Incorporating these systems into dryland carbon and nitrogen budgets may significantly revise estimates of microbial productivity in hyperarid zones (Couradeau et al. 2019; Weber et al. 2022).

Finally, as climate change alters fog regimes and coastal aridity patterns (Del Río 2020), the stability of these crust ecosystems faces new uncertainty. Any change in fog frequency or duration could curtail the delicate balance that sustains the grit crust communities, potentially shifting the system toward low‐biomass, opportunistic assemblages including bryophytes. Long‐term monitoring of these crusts thus holds importance not only for understanding Atacama Desert ecology but also for predicting the resilience of fog‐dependent ecosystems worldwide.

## 5 Conclusions

The microbial communities of the grit crust of the National Park Pan de Azúcar represent a unique intersection of productivity, specialisation, and resilience within the global spectrum of desert biocrusts. Their composition—dominated by *Trebouxia*‐lichen symbioses and proteobacterial associates—reflects adaptation to fog‐driven hydration and extreme oligotrophy. To date, this coherent, layer‐forming crust type has not been documented elsewhere; the most comparable system, in the Namib Desert, differs fundamentally in that phototrophs colonise the ventral surfaces of discrete quartz stones rather than forming a continuous biocrust, underscoring the singularity of the Atacama grit crust. The detected contrasts between black and white crusts encapsulate the functional continuum from early, dispersal‐driven stages to mature, symbiotic lichen holobiome communities. Having now resolved this homogeneous community pattern, future work employing metatranscriptomics and metagenomics will be essential to bring the functional roles and interdependencies of individual taxa into sharper focus. In this context, the grit crust provides an invaluable baseline for future assessments of disturbance recovery and for broader models of microbial life under extreme environmental constraints.

## Author Contributions

**Patrick Jung:** conceptualization, writing – original draft, funding acquisition, investigation, methodology, project administration, data curation, supervision. **Laura Briegel‐Williams:** writing – review and editing. **Karen Baumann:** writing – review and editing. **Lina Werner:** methodology, visualization, writing – review and editing. **Michael Lakatos:** resources, investigation, writing – review and editing, validation, supervision. **Rebekah Brand:** writing – review and editing, formal analysis, software, data curation. **Guillaume Letendu:** writing – review and editing, validation, formal analysis, software, data curation, methodology.

## Funding

This work was supported by Deutsche Forschungsgemeinschaft (JU 3228/1‐1), Bundesministerium für Bildung und Forschung (03WIR4502A, W2V‐Strategy2Value, 03WIR4516A, 03WIR4505B), Ministry of Science and Health Rhineland‐Palatinate (724‐0079#2024/0004‐1501 15404) and Schweizerischer Nationalfonds zur Förderung der Wissenschaftlichen Forschung (182531).

## Conflicts of Interest

The authors declare no conflicts of interest.

## Supporting information


**Table S1:** Manual corrections on the dataset based on information given from isolated organisms.
**Table S2:** Local ASV distribution.

## Data Availability

Metabarcoding data were submitted to the European Nucleotide Archive (ENA) under the project code PRJEB72845 https://www.ebi.ac.uk/ena/browser/view/PRJEB72845.
