# Supplementary material for: Fog, Symbiosis, and Survival: The Ecological Architecture of the Grit Crust From the Atacama Desert Represents a Lichen Holobiome Rather Than a Soil Microbiome
Source: Environ Microbiol. 2026 Jun 18;28(6):e70350. doi: 10.1111/1462-2920.70350 (PMC13278822; doi:10.1111/1462-2920.70350)
Supplement: Supplementary file 1 — Table S1: Manual corrections on the dataset based on information given from isolated organisms. Table S2: Local ASV distribution. [file EMI-28-e70350-s001.docx]

**Supplementary material**

**Table S1: Manual corrections on the dataset based on information given from isolated organisms.**

| **Number of ASVs** | **Total reads** | **Highest taxonomic assignment** | **Reference Database** | **Match; Accession Number; % ID** | **Best manual match; % ID** | **Final taxonomic assignment** | **Reason** | **Reference** |
| --- | --- | --- | --- | --- | --- | --- | --- | --- |
| 7 | 7023 | *Haliplanktos* sp. | SILVA | *Haliplanktos anthonyquinnii* TAU-MAC; 3018 NR_176599; 98% | Chroococcidiopsidales cyanobacterium KC20; OR713190; 100% | Chroococcidiopsidales sp. | New, yet undescribed genus within Chroococcidiopsidales | Jung et al. 2025 |
| 28 | 4801 | *Sinocapsa* sp. | SILVA | *Sinocapsa zengkensis* CHAB6751; MK567955; 81% | Chroococcidiopsidales cyanobacterium KC14; OR713213; 100% | Chroococcidiopsidales sp. | New, yet undescribed genus within Chroococcidiopsidales | Jung et al. 2025 |
| 23 | 3422 | uncultured Bacterium | SILVA | Uncultured Pleurocapsales cyanobacterium clone 8CYA_7; FJ985817; 100% | Chroococcidiopsidales cyanobacterium KC14; OR713213; 100% | Chroococcidiopsidales sp. | New, yet undescribed genus within Chroococcidiopsidales | Jung et al. 2025 |
| 2 | 5496 | *Myxosarcina* sp. | SILVA | *Myxosarcina* sp. SAG 30.84; KM019956; 99% | Chroococcidiopsidales cyanobacterium KC20; OR713190; 100% | Chroococcidiopsidales sp. | New, yet undescribed genus within Chroococcidiopsidales | Jung et al. 2025 |
| 1 | 796 | *Hyella* sp. | SILVA | *Hyella patelloides* LEGE 07179; HQ832901; 89% | Pleurocapsales cyanobacterium PJS31; MN266262; 100% | Plerocapsaceae cyanobacterium | New,yet undescribed genus within Pleurocapsaceae | Jung et al. 2021; Jung et al. 2025 |
| 63 | 353856 | *Acarospora* sp. | UNITE | *Acarospora* sp. PJ-2019a; LR584986; 97-100% | *Acarospora* sp. PJ-2019a; LR584986; 97-100% | *Acarspora conafii* | Only species of the area | Jung et al. 2019 |
| 69 | 129957 | *Amandinea* sp. | UNITE | *Amandinea* sp. isolate ALV8564; MN586921; 96% | Caliciaceae mycobiont FA5; OR726529; 100% | Caliciaceae | Defined lichen population | Jung et al. 2024 |
| 15 | 2418 | *Buellia almeriensis* | UNITE | *Buellia almeriensis* F5; MF062520; 87-92% | Caliciaceae mycobiont FA3; OR726506; 100% | Caliciaceae | Defined lichen population | Jung et al. 2024 |
| 24 | 20032 | Buellia decedens | UNITE | *Buellia decedens* 0197; DQ849319; 90-96% | Caliciaceae mycobiont FCF1; OR726514; 100% | Caliciaceae | Defined lichen population | Jung et al. 2024 |
| 26 | 127635 | *Buellia taishanensis* | UNITE | *Buellia taishanensis* TS1745; MG250190; 90-91% | Caliciaceae mycobiont FKCC2; OR726536; 100% | Caliciaceae | Defined lichen population | Jung et al. 2024 |
| 1181 | 664754 | *Pseudocyphellaria perpetua* | PR2 | *Pseudocyphellaria perpetua*; MH887529; 87-90% | Caliciaceae mycobiont FC1; OR726516; 100% | Caliciaceae | No cyanolichens occur in the habitat; very low proportion of *Nostoc* in the 16S dataset detected | Jung et al. 2024 |
| 77 | 744975 | Microthamniales | PR2 | *Trebouxia aggregata* SAG 219-1d; EU123942; 97% | *Trebouxia* sp. 17D2; OR726456; 100% | *Trebouxia* sp. | Manual correction | Jung et al. 2024 |
| 122 | 32600 | Microthamniales | PR2 | *Diplosphaera chodatii* SAG 11.88; MT078177; 96% | *Diplosphaera* sp. C3; PP079688; 100% | *Diplosphaera* sp. | Manual correction | Jung et al. 2024 |

**Table S2: Local ASV distribution.**

|  | **18S** |  |  | **16S** |  |  | **ITS** |  |  |
| --- | --- | --- | --- | --- | --- | --- | --- | --- | --- |
| **Site** | **ASVs [%] Black** | **ASVs [%] White** | **ASVs [%] shared** | **ASVs [%] Black** | **ASVs [%] White** | **ASVs [%] shared** | **ASVs [%] Black** | **ASVs [%] White** | **ASVs [%] shared** |
| 1 | 24 | 10 | 66 | 11 | 28 | 60 | 55 | 25 | 20 |
| 2 | 39 | 20 | 41 | 74 | 17 | 9 | 70 | 13 | 17 |
| 3 | 51 | 21 | 28 | 66 | 30 | 4 | 68 | 12 | 21 |
| 4 | 28 | 35 | 37 | 34 | 51 | 15 | 70 | 16 | 14 |
| 5 | 24 | 32 | 44 | 32 | 45 | 23 | 58 | 23 | 19 |
| 6 | 26 | 28 | 46 | 66 | 11 | 24 | 36 | 22 | 42 |
| 7 | 3 | 92 | 6 | 18 | 73 | 9 | 15 | 73 | 12 |
| 8 | 36 | 26 | 38 | 45 | 39 | 16 | 50 | 19 | 32 |
| 9 | 27 | 34 | 39 | 28 | 48 | 24 | 57 | 14 | 30 |
| 10 | 25 | 26 | 49 | 65 | 20 | 16 | 58 | 16 | 26 |
| 11 | 39 | 31 | 31 | 63 | 29 | 8 | 55 | 14 | 31 |
| **Mean** | 29.2 ± 12.4 | 32.2 ± 21.1 | 36.6 ± 14.8 | 45.6 ± 22.1 | 35.4 ± 18.0 | 19.0 ± 15.3 | 53.7 ± 16.3 | 22.2 ± 17.5 | 24.1 ± 8.9 |
